# BCIP: a gene-centered platform for identifying potential regulatory genes in breast cancer

**DOI:** 10.1038/srep45235

**Published:** 2017-03-22

**Authors:** Jiaqi Wu, Shuofeng Hu, Yaowen Chen, Zongcheng Li, Jian Zhang, Hanyu Yuan, Qiang Shi, Ningsheng Shao, Xiaomin Ying

**Affiliations:** 1 Beijing Institute of Basic Medical Sciences, Beijing 100850, China; 2 Department of Obstetrics and Gynecology, Fuzhou General Hospital of Nanjing Military Command, Fuzhou, Fujian 350025, China; 3 Translational Medicine Center of Stem Cells, 307-Ivy Translational Medicine Center, Laboratory of Oncology, Affiliated Hospital, Academy of Military Medical Sciences, Beijing 100071, China

## Abstract

Breast cancer is a disease with high heterogeneity. Many issues on tumorigenesis and progression are still elusive. It is critical to identify genes that play important roles in the progression of tumors, especially for tumors with poor prognosis such as basal-like breast cancer and tumors in very young women. To facilitate the identification of potential regulatory or driver genes, we present the Breast Cancer Integrative Platform (BCIP, http://omics.bmi.ac.cn/bcancer/). BCIP maintains multi-omics data selected with strict quality control and processed with uniform normalization methods, including gene expression profiles from 9,005 tumor and 376 normal tissue samples, copy number variation information from 3,035 tumor samples, microRNA-target interactions, co-expressed genes, KEGG pathways, and mammary tissue-specific gene functional networks. This platform provides a user-friendly interface integrating comprehensive and flexible analysis tools on differential gene expression, copy number variation, and survival analysis. The prominent characteristic of BCIP is that users can perform analysis by customizing subgroups with single or combined clinical features, including subtypes, histological grades, pathologic stages, metastasis status, lymph node status, *ER/PR/HER2* status, *TP53* mutation status, menopause status, age, tumor size, therapy responses, and prognosis. BCIP will help to identify regulatory or driver genes and candidate biomarkers for further research in breast cancer.

Breast cancer is a frequently diagnosed carcinoma and is the leading cause of cancer death among females worldwide. An estimated 1,676,600 cases were diagnosed and 521,900 deaths occurred in 2012, accounting for 25% of the total cancer cases and 15% of all the cancer deaths among females^1^. Breast cancer is a heterogeneous disease with a high degree of diversity in morphology, histology, pathological features, and molecular alterations, such as gene mutations and abnormal expression^2^. Researches based on gene expression (GE) patterns have classified breast cancer into distinct subgroups corresponding to different prognostic outcomes and therapeutic responses^3^,^4^,^5^,^6^. A number of studies have focused on the recognition of biomarkers and characterization of gene function in particular breast cancer subgroups^7^,^8^,^9^,^10^.

Triple-negative breast cancer (TNBC) is a highly aggressive subtype of breast cancer and the vast majority is basal-like phenotype^11^. Due to its high genetic heterogeneity, TNBC does not possess a common genetic mutation and thus lacks effective targeted therapies^12^. However, several studies based on GE have identified several critical genes that may be potential druggable targets for the treatment of TNBC^13^,^14^,^15^. For example, *MELK* has been characterized as an oncogenic kinase essential for basal-like breast cancer (BBC) via a kinome-wide screening, integrative analysis with multiple GE datasets, and further *in vitro* and *in vivo* experiments^14^. *BCL11A* has also been reported to be a novel TNBC oncogene by *in silico* analysis of several microarray datasets and subsequent experimental validations^15^. These studies suggest that GE profiles are important resources for regulatory gene and biomarker identification in breast cancer. Differential expression analysis, copy number variation (CNV) analysis, survival analysis, and co-expression analysis on multiple credible and qualified datasets are effective approaches for recognizing novel regulatory genes and biomarkers.

In order to help researchers use gene expression profiles, some databases and tools have been developed^16^,^17^,^18^,^19^. However, integrative platforms combined with multi-omics data and customized analysis tools for breast cancer are still lacking. In this study, we developed BCIP, which provides differential expression analysis, copy number variation analysis, survival analysis, co-expression analysis, microRNA (miRNA) regulation analysis, and pathway analysis for query genes. To ensure the reliability of the analysis, we collected and obtained GE profile data on 9,381 samples from 29 datasets with strict quality control and uniform processing. We also incorporated CNV information on 3,035 samples, 324,219 miRNA-target interactions, 286 KEGG pathways, and data from tissue-specific gene functional networks of mammary gland and mammary epithelium. In order to facilitate researchers’ analysis of the specific subgroups they focused on, we developed a comprehensive and flexible interface that permits users to customize subgroups with single or combined clinical features of interest, including subtypes, grades, stages, metastasis status, lymph node status, prognosis, age, tumor size, *ER/PR/HER2* status, *TP53* mutation status, menopause status, and therapy response. BCIP will be a valuable tool for the identification of regulatory or driver genes in breast cancer.

## Methods

### Data collection and processing

We initially retrieved and collected data from NCBI Gene Expression Omnibus^20^ (GEO), European Genome-phenome Archive of EMBL European Bioinformatics Institute^21^ (EMBL-EBI), and The Cancer Genome Atlas^22^ (TCGA) with the following criteria: (1) gene microarray or high-throughput sequencing data of RNAs extracted from primary breast tumor or adjacent normal tissues; (2) the sample size of each dataset is no less than 50; (3) clinical information were provided together with the dataset, mainly including subtypes, histological grades, pathologic stages, ER/PR/HER2 status, and prognosis; (4) the dataset was available for download before Jan 1, 2016, which was the latest date we collected the datasets. Finally, we obtained a preliminary collection of 86 independent datasets. To assure adequate specimens in subgrouping, we assessed the sample number demand and removed the datasets with less than 100 samples. The rest 30 datasets include 27 datasets from GEO (measured by Affymetrix microarray), 2 datasets from TCGA (measured by Agilent microarray and Illumina HiSeq), and 1 dataset of the METABRIC from EBI (measured by Illumina HT-12 v3 microarray).

Then we performed quality control, normalization and duplicate removing on all the 30 datasets. Quality control was carried out by *simpleaffy* and *affyPLM* R packages on each of the 27 GEO datasets independently. The raw data of each dataset were then normalized, summarized, and log-transformed using robust multi-array average (RMA) function of *affy* R package. The probe-based expression was converted into GE profiles, and the gene containing multiple probes was represented by the probe with the largest interquartile range across the samples. For the METABRIC dataset, we deleted 12 samples since 8 samples were duplicated in the discovery and validation sets and 4 were represented twice in the validation set. We used the processed expression matrix data of METABRIC directly^23^. For the TCGA Agilent and RNA-Seq data, we removed 22 samples without matched clinical information and used level_3 log2 normalized data from TCGA directly.

Furthermore, the tumor purity of the samples profiled on Affymetrix platforms was detected through a robust method, ESTIMATE, which uses the ESTIMATE-based tumor purity score developed by Affymetrix data to evaluate tumor purity^24^. This method was not applied to predict the tumor purity of the samples profiled on Affymetrix Human Genome U133B Array because of the insufficiency of the gene signatures intersection. Depending on the results of tumor purity estimation (Supplementary Figure S1), we eliminated one dataset with the lowest mean tumor purity, in order to reduce noises caused by diverse tumor purity. Finally, GE profiles of a total of 26,339 genes from 9,381 samples of 29 datasets were available for transcriptome analysis.

### Sample subgrouping features

We compiled a series of clinical features along with each sample for sample subgrouping. For samples with some clinical features that were not initially provided (mainly *ER*^−/+^/*PR*^−/+^/*HER2*^−/+^, TNBC, and PAM50 subtypes) in certain collected datasets, we defined these features using a computational method based on GE profiles. Expressions of *ER, PR*, and *HER2* were respectively fitted by a Gaussian bimodal distribution model and the parameters were estimated via EM algorithm using *Mclust* function in *mclust* R package. The expression status for *ER, PR*, and *HER2* were discriminated as positive (*ER*^+^, *PR*^+^, and *HER2*^+^) or negative (*ER*^−^, *PR*^−^, and *HER2*^−^). On the basis of the identification of *ER, PR*, and *HER2* positive or negative expression status, we classified samples into TNBC or non-TNBC subtype^6^. Samples defined as *ER*^−^, *PR*^−^ and *HER2*^−^ status were identified as TNBC and otherwise non-TNBC. Molecular classification for PAM50 subtypes was provided in some datasets, and if not, we classified the patients into the five intrinsic breast cancer subtypes using the 50-gene subtype classifier, PAM50^4^. The feature of the prognosis status was classified into good/poor prognosis using the median survival time as the delimitation.

### Copy number variation

We obtained CNV data for 28,678 genes of 3,035 samples from METABRIC and TCGA. Both the DNA microarray platforms of the 2 datasets are Affymetrix Genome-Wide Human SNP Array 6.0. The numerical values of CNV were processed, summarized, and normalized relying on the relative intensity of probe hybridization on the arrays. Segmented data were converted to the gene level matrix using GISTIC 2.0^25^, which were annotated for gene content based on hg19/GRCh37 for the TCGA data. For the METABRIC data, we generated a patient-by-gene CNV matrix through the processed segment data by matching the overlap of the segments with the gene regions whose annotations and coordinates were given by hg18/Ensembl 54. For more accurate and reliable analysis, we set the gain/loss threshold to 0.1 and −0.1, respectively. When the CNV value of a gene is greater than 0.1, the gene is defined as copy number gain. When the CNV value of a gene is smaller than −0.1, the gene is defined as copy number loss.

### Statistical analysis

All the statistical analysis were performed using R programming platform. An unpaired *t* test was used for differential GE analysis in Transcriptome Analysis for 2 subgroups. One-way analysis of variance (ANOVA) was used for more than 2 subgroups if GE satisfied the assumption of a normal distribution, and if not, the non-parametric test (Kruskal–Wallis test) was used to assess statistical differences among these subgroups. The *survfit* function of *survival* R package was used for survival analysis. Kaplan-Meier curves and log-rank test were used to assess survival differences. In Transcriptome Survival Analysis, we classified patients into 2 groups according to an optimal GE cutoff value based on the Cutoff Finder application^26^. This program will traverse the GE values of all patients and the optimal cutoff value can minimizes the p value of survival differences. In CNV Survival Analysis, patients are separated into 2 groups according to their CNV status (gain/loss) of the query gene. Hazard ratio (HR) was calculated using Cox proportional hazards regression model. Co-expression analysis was performed using *cox* function of *WGCNA* R package. The correlation of GE was evaluated by Pearson correlation coefficient (PCC) as well as false discovery rate (FDR) adjusted p-value. The genes with absolute PCC ≥ 0.3 and adjusted p-value ≤ 0.05 were considered co-expressed in Co-expression Analysis.

### Database schema and implementation

BCIP was implemented based on the Apache HTTP server 2.2 with MySQL 5.1.73 at the back end and the PHP 5.5.31, HTML, and JavaScript at the front end. All the computing programs were completed with R 3.2.3 and dependent packages.

## Results

### Overview of BCIP

**Figure 1:**
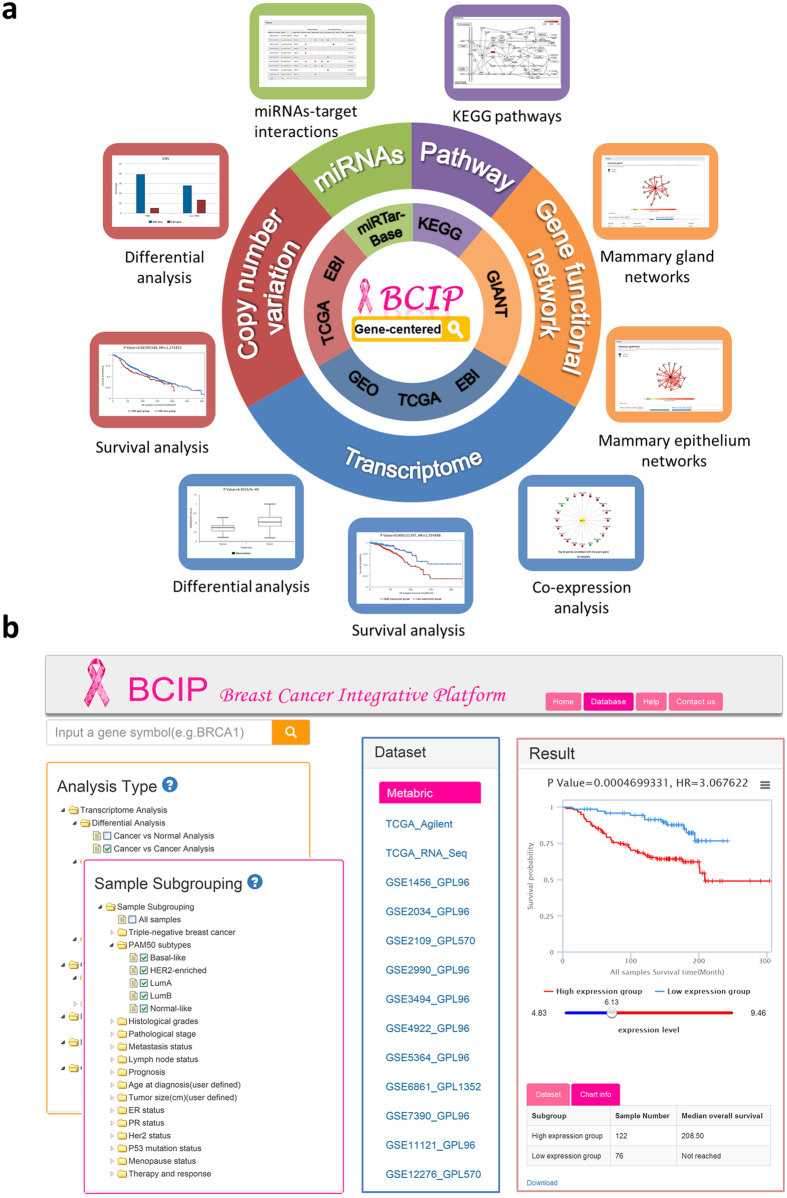
Schematic diagram showing the architecture of BCIP. (**a**) Data sources and applications of BCIP. The same color system was used to characterize the correspondence, while the inner light-colored donut chart represents the data sources and the outer deep-colored donut chart indicates the corresponding applications (analysis types). (**b**) Overview of the data portal of BCIP. The panel of 4 modules includes: *Analysis Type, Sample Subgrouping, Dataset,* and *Result*.

BCIP is a gene-centered platform that provides (1) differential expression analysis, survival analysis, and co-expression analysis based on transcriptome data; (2) differential analysis and survival analysis based on CNVs; (3) miRNA regulation analysis on miRNA-target interactions; (4) KEGG pathway analysis; and (5) network analysis on mammary tissue-specific gene function networks (Fig. 1a). BCIP provides a user-friendly interface consisting of four panels: *Analysis Type, Sample Subgrouping, Dataset*, and *Result* (Fig. 1b). A gene symbol can be input in the text field where we provide a fuzzy matching function. Users can then select any of 5 analytical categories in the *Analysis Type* panel, including Transcriptome Analysis, Copy Number Variation Analysis, MicroRNA-target Interaction Analysis, Pathway Analysis, and Gene Functional Network Analysis. After selecting analytical category, users can customize subgroups with single or combined clinical features of interest in the *Sample Subgrouping* panel. BCIP provides a total of 15 clinical features, including TNBC and non-TNBC subtypes, PAM50 subtypes, histological grades, pathologic stages, metastasis status, lymph node status, *ER*/*PR*/*HER2* status, *TP53* mutation status, menopause status, age, tumor size, therapy responses, and prognosis. The *Dataset* panel provides all of the available datasets for the selected options in the *Analysis Type* and *Sample Subgrouping*. Finally, the *Result* panel returns corresponding graphical and tabular presentation and analysis results after choosing from the above options.

### Transcriptome Analysis

**Figure 2:**
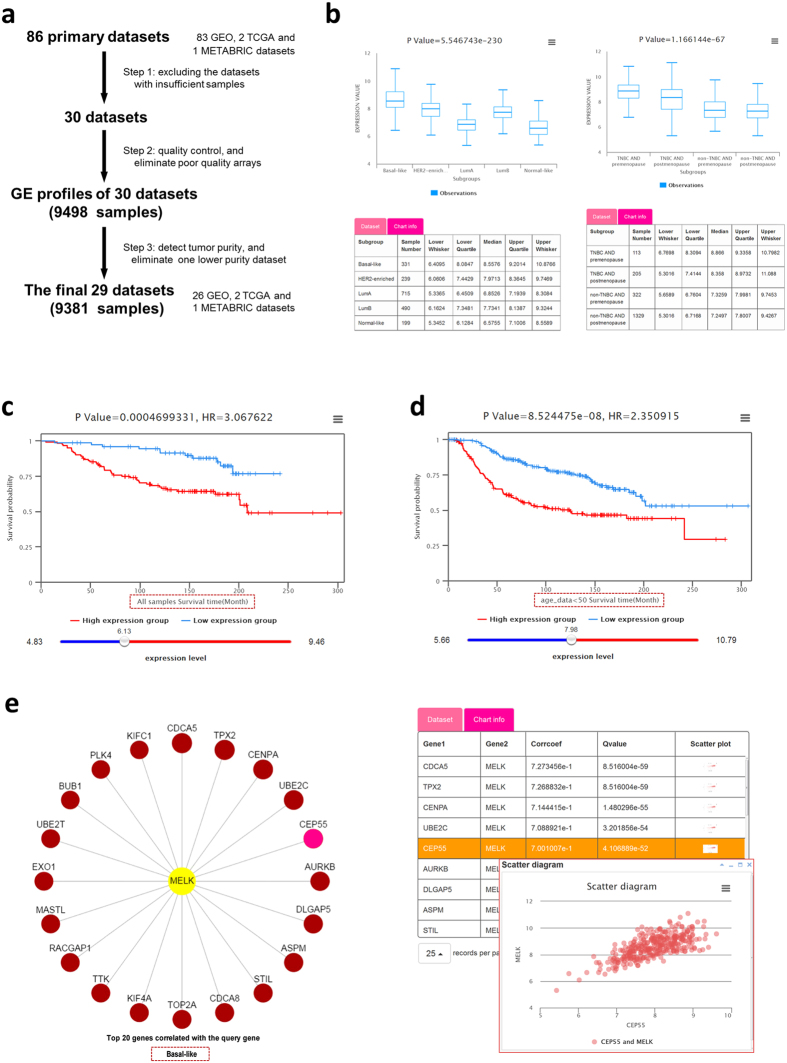
Visualization of transcriptome data information and analytic results. (**a**) Flow chart of the transcriptome data processing procedures. (**b**) Box plots showing differential gene expression of *MELK* in breast cancer PAM50 subtypes (left) and other subgroups (right): TNBC AND premenopause, TNBC AND postmenopause, non-TNBC AND premenopause, and non-TNBC AND postmenopause. The tables display detailed information of the dataset and each subgroup of the chart. (**c**,**d**) The low (blue curve) and high (red curve) levels of *MELK* expression groups are correlated with the overall survival times in all patients of the GSE7390 dataset (p-value = 0.00047, HR = 3.067622) or in the patients younger than 50 years old of the METABRIC dataset (p-value = 8.524475e-08, HR = 2.350915). (**e**) The circle dots show the top 20 genes co-expressed with *MELK* in basal-like subtype patients of the METABRIC dataset. Positive and negative correlation is respectively distinguished by red and green circles. The table lists co-expressed genes with PCC ≥ 0.3 and adjusted p-value ≤ 0.05. Scatter plot showing the expression status of *MELK* and *CEP55*.

We collected GE data of breast cancer tissue samples from publicly available databases of GEO, EMBL-EBI and TCGA and obtained 86 datasets. After excluding the datasets with insufficient samples (less than 100) or low tumor purity, we finally retained 29 datasets with the GE profiles of 9,381 samples (Fig. 2a and Supplementary Table S1). The GE profiles are used for differential expression analysis, survival analysis, and co-expression analysis.

- **Differential expression analysis.** There are 2 options for differential expression analysis in *Analysis Type: cancer vs normal, cancer vs cancer*. The *cancer vs normal* option is designed to show the expression difference between the tumor and normal tissues. The *cancer vs cancer* option supports differential analysis in tumor samples among user-defined subgroups. Users are allowed to customize specific subgroups with single or combined clinical features of interest. Differential expression analysis results will be illustrated with a box plot in the *Result* panel. Dataset and chart information are also presented below the graph. For example, *MELK*, a recently reported oncogenic kinase in BBC^14^, has the highest expression level in basal-like subtype in the METABRIC dataset (Fig. 2b, left panel). When both TNBC and non-TNBC in the Triple-negative breast cancer group and pre-menopause/post-menopause in the Menopause status group are selected, BCIP will divide samples into 4 subgroups according to the combination of 2 groups (Fig. 2b, right panel).

- **Survival analysis.** Survival analysis is provided to investigate the association of gene with clinical prognosis. BCIP offers 5 survival types, including overall survival (OS), disease-specific survival (DS), disease-free survival (DFS), recurrence-free survival (RFS), and distant metastasis-free survival (DMFS). Users can perform survival analysis in the specific subgroup customized with single or combined clinical features of interest. Patients in the specific subgroup are divided into 2 groups according to an optimal cutoff of the GE levels. The optimal cutoff is determined by the Cutoff Finder that maximizes the survival differences between 2 groups^26^. Notably, the cutoff value can be flexibly moved through a slider bar. Kaplan-Meier survival curves will be redrawn dynamically with the change of the cutoff value, together with the p-value and HR. Figure 2c and d show the overall survival analysis results of *MELK* in all patients of the GSE7390 dataset and in the samples younger than 50 years old of the METABRIC dataset, respectively.
- **Co-expression analysis.** Co-expression analysis is widely used to provide clues for potential associations among genes, proteins, and other biomolecules in various carcinomas^27^,^28^,^29^. Given a gene, BCIP displays the top 20 co-expression genes in the form of a circle dot where positive and negative correlations are represented by red and green, respectively (Fig. 2e). All of the co-expression genes with absolute PCC ≥ 0.3 and adjusted p-value ≤ 0.05 are presented in descending order of PCCs in the table below the graph. Scatter plots of GE levels of the co-expressed genes and the query gene are provided when clicking on the dots or the last column of the table. Notably, BCIP permits users to investigate co-expression relationship in any specified subgroup that users customized with the clinical features of interest.

### Copy Number Variation Analysis

CNVs exist pervasively in human genomes and contribute to the diversity and susceptibility of numerous diseases^30^. It may be an important factor in cancer occurrence and development. A series of studies and attempts have been carried out to explore the impact of CNV on breast cancer^31^,^32^,^33^. We collected and incorporated CNVs information of 3,035 tumor samples from METABRIC and TCGA. BCIP provides differential analysis and survival analyses for CNV data.

**Figure 3:**
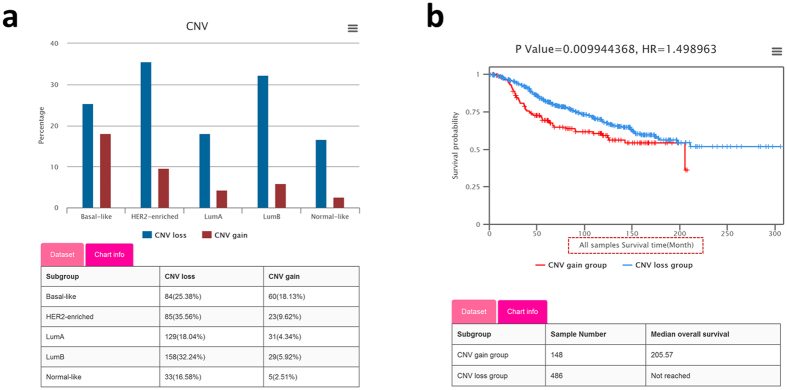
Visualization of copy number variations (CNVs) based analyses reveals CNV status and survival correlation. (**a**) Histogram depicting the percentage of samples with *MELK* copy number losses/gains in each PAM50 subtype of the METABRIC dataset. (**b**) Kaplan-Meier plot showing the disease-specific survival rate comparison between patients with *MELK* copy number losses (blue curve) and copy number gains (red curve), and the tables showing the sample numbers and median survival times of the 2 groups.

- **Differential analysis** **between copy number gain/loss.** The proportions of samples with CNV (copy number gain and loss) of the query gene in any user-defined subgroups are respectively displayed in a histogram. A table matching the histogram provides details of the sample numbers and the proportions of copy number gain/loss in the corresponding subgroups. For example, *MELK* has a much higher (more than 2 times) proportion of samples with copy number gain in basal-like subtype than other PAM50 subtypes (Fig. 3a), which provides a clue to explain its high expression in BBC.
- **Survival analysis with CNV data.** Survival analysis based on gene CNVs will shed light on the correlation between prognostic outcomes and CNV status. BCIP provides 5 survival types (OS, DS, DFS, RFS, and DMFS), permitting users to perform analysis in specific subgroups customized with single or combined clinical features of interest. Samples are separated into 2 groups according to their CNV status (gain/loss) of the query gene. The Kaplan-Meier plot shows that the samples with *MELK* copy number gain have shorter disease-specific survival times than those with copy number loss in the METABRIC dataset (Fig. 3b).

### MicroRNA-target Interaction Analysis

miRNAs are small non-coding RNAs that can regulate protein-coding messenger RNAs (mRNAs) at the post-transcriptional level^34^. The pivotal role of miRNAs is known as a modulator participating in various biological processes. Numerous studies suggest that dysregulation of miRNAs may contribute to the initiation and progression of cancers^35^, and miRNAs can be regarded as diagnostic signatures or therapeutic biomarkers in breast cancer^36^,^37^. To facilitate researchers’ investigations into potential regulation mechanisms of query genes, BCIP provides MicroRNA-target Interaction Analysis, which illustrates miRNAs targeting the query gene. There is a total of 324,219 miRNA-target interactions between 2,619 miRNAs and 14,884 target genes from miRTarBase^38^ that are maintained in BCIP. All of these interactions are experimentally validated.

**Figure 4:**
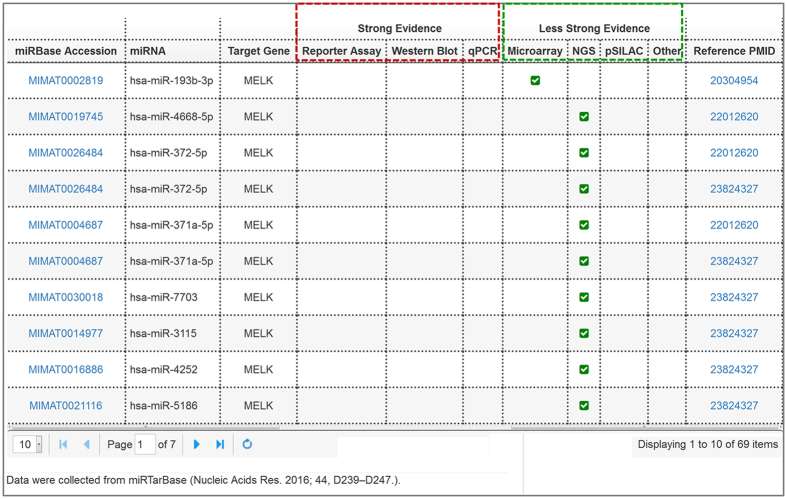
The miRNA-target interactions table lists the experimentally validated mature miRNAs that target *MELK*. The red box and green box respectively represent the strong experiment evidence (reporter assay, Western blot, and qPCR) and less strong evidences (microarray and next-generation sequencing experiments).

- **miRNA-target interactions.** The results for miRNA-target interactions analysis are presented as a table and list the mature miRNAs that target the input gene and the corresponding experiment types (Fig. 4). Experiment types of reporter assay, Western blot, real-time quantitative PCR (qPCR) are regarded as strong evidence, while microarray, next-generation sequencing (NGS), and pulsed stable isotope labeling by amino acids in cell culture (pSILAC) are regarded as less strong evidence for the interactions between miRNAs and genes^38^. In addition, external links to the miRBase database and PubMed have been embedded in the table for the added convenience in retrieving corresponding information.

### Pathway Analysis

It is known that some biological pathways involved in metabolism, apoptosis, and signal transduction play critical roles in cell proliferation and differentiation during tumorigenesis and cancer development. Understanding which pathways a gene participates in will be of great help for researchers in characterizing its functions in breast cancer. We have collected 22,455 linked entries between 6,755 genes and 286 human pathways from the KEGG database^39^.

**Figure 5:**
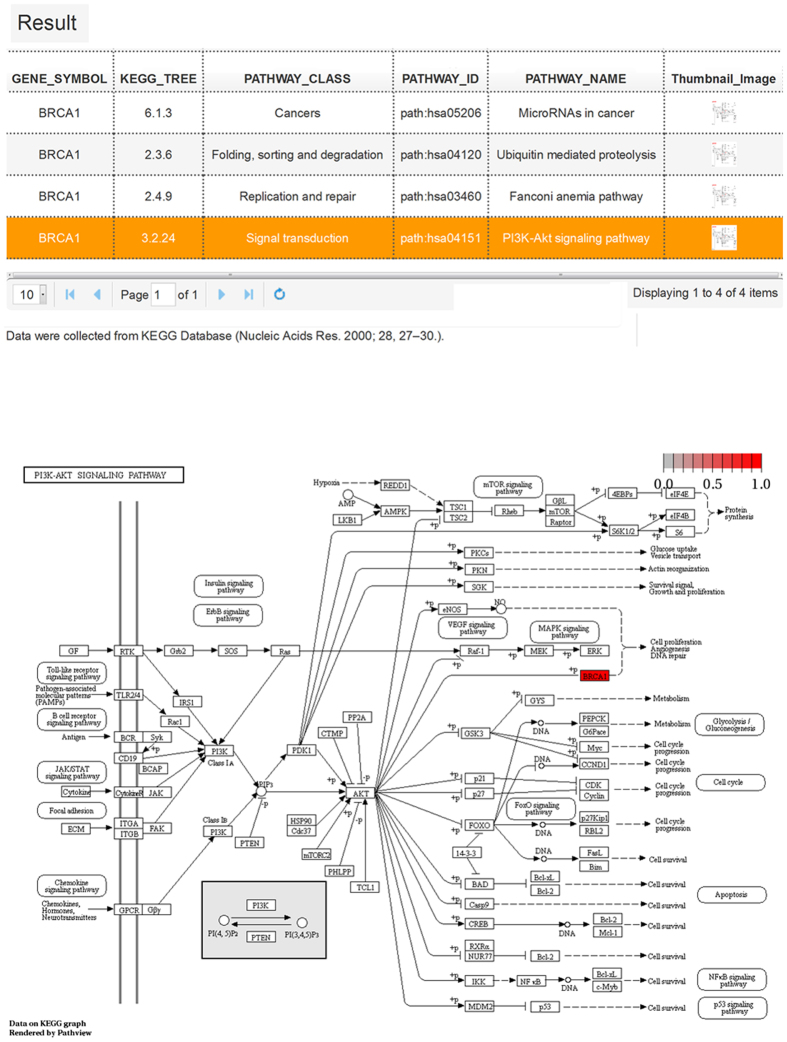
KEGG pathway analysis results and pathway map visualization of *BRCA1*. The table lists all of the pathways involving gene *BRCA1*, which consists of the KEGG tree, pathway class, pathway ID, and pathway name. The schematic representation showing the PI3K-Akt signaling pathway map^39^, which depicts the molecular interaction and reaction network. Gene *BRCA1* is highlighted in red.

- **KEGG pathways.** A table consisting of pathway classes, pathway IDs, and pathway names are presented in the KEGG pathways analysis results to depict pathway information (Fig. 5). The thumbnail images in the last column can pop-up pathway maps when clicked. The pathway maps contain molecular interaction and reaction networks in which the query gene was involved. The query gene is highlighted in red.

### Gene Functional Network Analyses

Each biomolecule is located in complex biological networks and exerts its functions together with other related molecules. Notably, gene expression as well as gene-gene functional relationships in the complex biological regulation network may be tissue-specific^18^,^40^. Identifying a gene’s functional partner in specified tissue can facilitate researchers to infer gene functions and molecular mechanisms. Here we provide mammary tissue-specific gene functional networks analysis. Data on both mammary epithelium and mammary gland gene functional networks were collected and processed from the GIANT webserver^40^.

**Figure 6:**
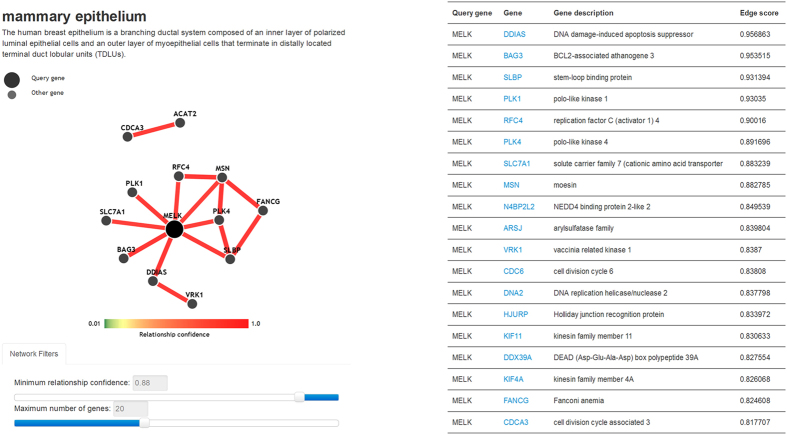
Visualization of the mammary epithelium-specific gene functional network. The dynamic network displays the front portion of the entire network of *MELK* with 50 other genes. The number of genes in the network is controlled by the slider bar of the minimum relationship confidence and the maximum number of genes. The table lists the top 50 related genes in accordance with the priority of average edge score.

- **Mammary epithelium and gland networks.** Users are allowed to search gene functional relationships in mammary epithelium and gland gene functional networks. The dynamic network shows a subset of the entire network, and the nodes and edges are controlled by the slider bars of the maximum number of genes and the minimum relationship confidence (Fig. 6). A table below the network lists the details of the top 50 functional related genes in descending order of the average edge score, which reflects the relationship strength between the 2 genes. Clicking on any gene leads to a new table showing the corresponding detail about that gene.

## Case Study and Discussion

For a query gene, BCIP helps to demonstrate its potential as a biomarker or regulatory gene in breast cancer. Take *MELK*, a promising therapeutic target of BBC reported recently, as an example to demonstrate the utility and advantage of BCIP^14^. Differential expression analysis shows that *MELK* has a much higher expression level in tumors than adjacent normal tissues across all of the available datasets (Supplementary Figure S2) and has the highest expression level in basal-like subtype among PAM50 subtypes across all of the datasets (Supplementary Figure S3). When subgroups are customized with tumor grades, we found that higher *MELK* expression level was significantly associated with higher histological grades among all of the datasets (Supplementary Figure S4). Survival analysis shows that overexpression of *MELK* is strongly correlated with poor prognosis (Supplementary Figure S5–9). These *in silico* results indicate that *MELK* might play roles in BBC.

To provide clues of the possible molecular mechanism of *MELK* in BBC, we further analyzed the co-expression genes and regulatory miRNAs of *MELK*. In basal-like subtype of the METABRIC dataset, *MELK* was significantly co-expressed with 78 genes with PCC > 0.6, including *CDCA5, TPX2*, and *CEP55* (Fig. 2e). Several studies have demonstrated that *TPX2* and *CEP55* are critical molecules for breast cancer migration, invasion, cell proliferation, and metastasis^41^,^42^,^43^. *CDCA5* has been reported to play a crucial role in human lung carcinogenesis and has the potential of being a therapeutic target for oral squamous cell carcinoma^44^,^45^. These results may be valuable clues for the investigation of potential function and molecular mechanism of *MELK* in breast cancer. Additionally, we found miRNAs targeting *MELK*, including hsa-miR-193b-3p and hsa-miR-372-5p (Fig. 4). Previous studies have shown that miR-193b represses cell proliferation and regulates cyclin D1 in melanoma^46^, and miR-372 suppresses tumor proliferation, invasion, and migration in various tumor types^47^,^48^. This result indicates that *MELK* might be regulated by miR-193b-3p and miR-372-5p in breast cancer.

We have developed BCIP, a user-friendly, open-access, integrative analysis platform that integrates almost 10,000 tumor and normal tissue samples of breast cancer. It will facilitate the identification of potential biomarkers and regulatory genes in breast cancer. Compared with other bioinformatics resources and analysis tools, BCIP has 3 unique characteristics: (i) BCIP incorporates multiple analysis types, including differential expression analysis, copy number variation and survival analysis, gene co-expression analysis, miRNA regulation analysis, KEGG pathways presentation, and mammary tissue-specific gene functional network analysis. All of these analysis tools help to sketch an overview of a gene in breast cancer. (ii) It provides dozens of datasets that are screened from publicly available databases, selected with strict quality control and processed with uniform normalization methods. Users can observe the consistency of the analysis results across multiple datasets, which will be helpful to evaluate the robustness of analysis results. (iii) BCIP permits users to perform analysis in specific breast cancer subgroups that are customized with single or combined clinical features of interest, including molecular subtypes, therapy response, and various clinical features.

Lots of studies have been done to identify biomarkers and to uncover molecular mechanisms of tumorigenesis, cell invasion, and metastasis in breast cancer. However, many tumors with high invasion and poor outcomes, such as TNBC or basal-like tumors, still lack well-defined molecular biomarkers and therapy targets due to the high heterogeneity. BCIP serves as a convenient and efficient platform to identify biomarkers, characterize potential functions and mechanisms of genes in breast cancer. Researchers can find clues for subsequent experiments and clinical analysis. In our future work, we will continue incorporating newly available, credible data into BCIP and provide reliable supports for researchers of breast cancer.

## Additional Information

**How to cite this article:** Wu, J. *et al*. BCIP: a gene-centered platform for identifying potential regulatory genes in breast cancer. *Sci. Rep.* **7**, 45235; doi: 10.1038/srep45235 (2017).

**Publisher's note:** Springer Nature remains neutral with regard to jurisdictional claims in published maps and institutional affiliations.

## Supplementary Material

Supplementary Information

Supplementary Table S1
