# Corrigendum: BCIP: A gene-centered platform for identifying potential regulatory genes in breast cancer

**DOI:** 10.1038/srep46913

**Published:** 2017-12-22

**Authors:** Jiaqi Wu, Shuofeng Hu, Yaowen Chen, Zongcheng Li, Jian Zhang, Hanyu Yuan, Qiang Shi, Ningsheng Shao, Xiaomin Ying

Scientific Reports 7: Article number: 45235; 10.1038/srep45235 published online: 03 22 2017; updated: 12 22 2017.

The original version of this Article contained an incorrect link to the Breast Cancer Integrative Platform in the Abstract.

“To facilitate the identification of potential regulatory or driver genes, we present the Breast Cancer Integrative Platform (BCIP, http://omics.bmi.ac.cn/bcancer/).”

now reads:

“To facilitate the identification of potential regulatory or driver genes, we present the Breast Cancer Integrative Platform (BCIP, http://www.omicsnet.org/bcancer/).”

This has now been corrected in the PDF and HTML versions of the Article.

