# Supplementary material for: BCIP: a gene-centered platform for identifying potential regulatory genes in breast cancer
Source: Sci Rep. 2017 Mar 22;7:45235. doi: 10.1038/srep45235 (PMC5361122; doi:10.1038/srep45235)
Supplement: Supplementary Information [file srep45235-s1.pdf]

Supplementary Information for:

BCIP: a gene-centered platform for identifying potential regulatory genes in breast cancer

Jiaqi Wu[1†], Shuofeng Hu[1†], Yaowen Chen[1,2], Zongcheng Li[1,3], Jian Zhang[1], Hanyu Yuan[1], Qiang Shi[1], Ningsheng Shao[1] and Xiaomin Ying[1*]

[1]Beijing Institute of Basic Medical Sciences, Beijing 100850, China
[2]Department of Obstetrics and Gynecology, Fuzhou General Hospital of Nanjing Military Command, Fuzhou, Fujian 350025, China
[3]Translational Medicine Center of Stem Cells, 307-Ivy Translational Medicine Center, Laboratory of Oncology, Affiliated Hospital, Academy of Military Medical Sciences, Beijing 100071, China

[*]Corresponding author:

Xiaomin Ying, PhD

Computational Omics Lab, Center of Computational Biology

Beijing Institute of Basic Medical Science

Beijing 100850, China

Tel: 86 10 6821 3039

Email: yingxm@bmi.ac.cn; yingxmbio@gmail.com

[†]These authors contributed equally to this work.

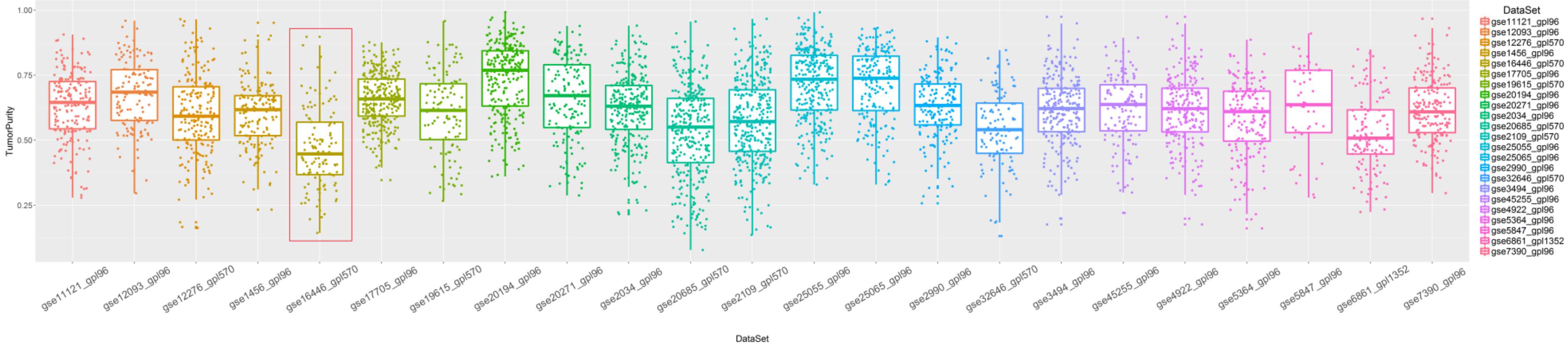

Supplementary Figure S1. The ESTIMATE-predicted tumor purity of samples in several datasets profiled on Affymetrix platforms. Box plots show the tumor purity of samples in 23 datasets. The results show that the mean tumor purity of a dataset, GSE16446, was obviously lower than that of other datasets.

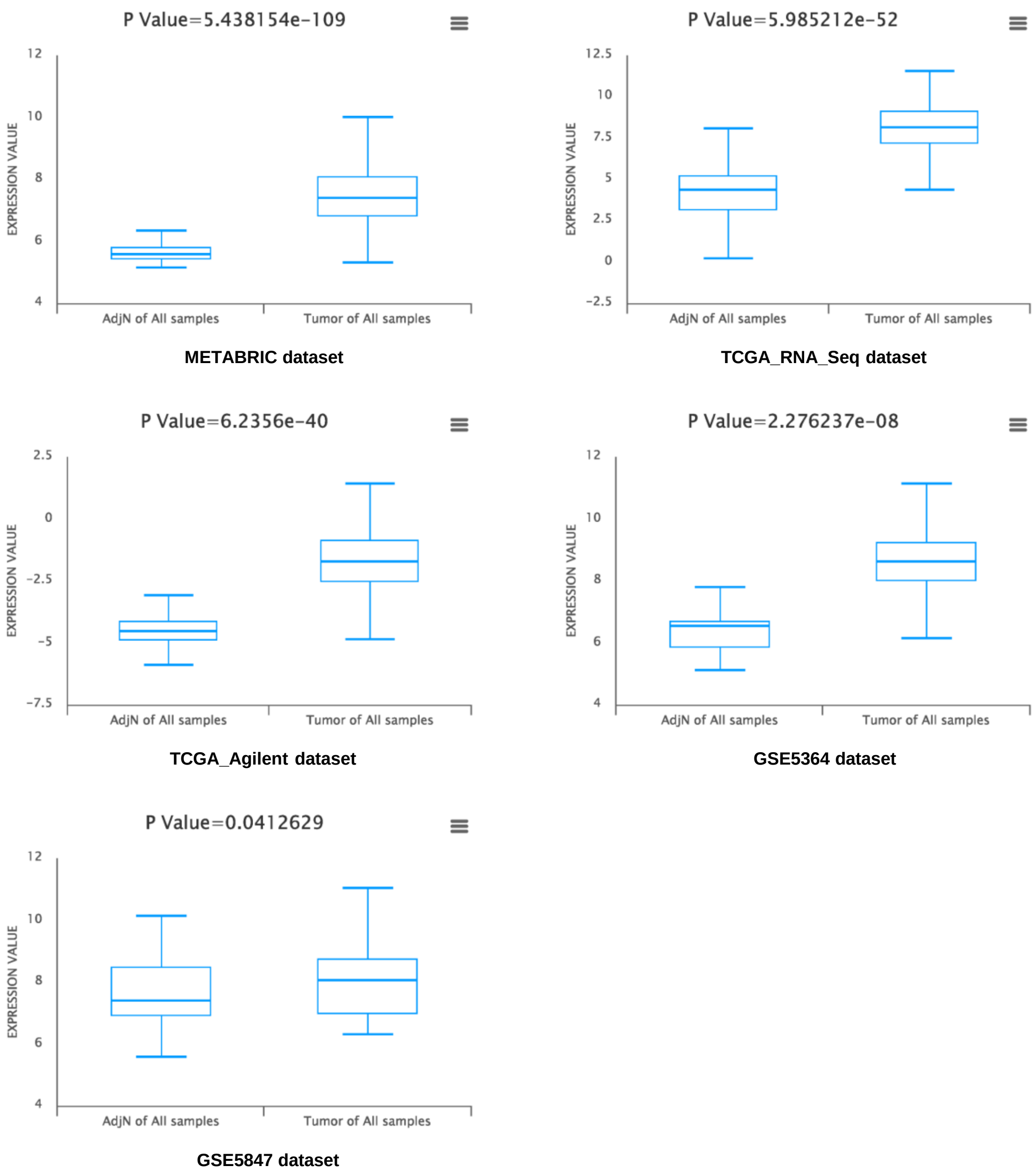

Supplementary Figure S2. Differential expression of *MELK* between breast tumors and adjacent normal tissues. Box plots show the differential expression of *MELK* between breast tumors and adjacent normal (AdjN) tissues in the METABRIC, TCGA_RNA_Seq, TCGA_Agilent, GSE5364, and GSE5847 datasets.

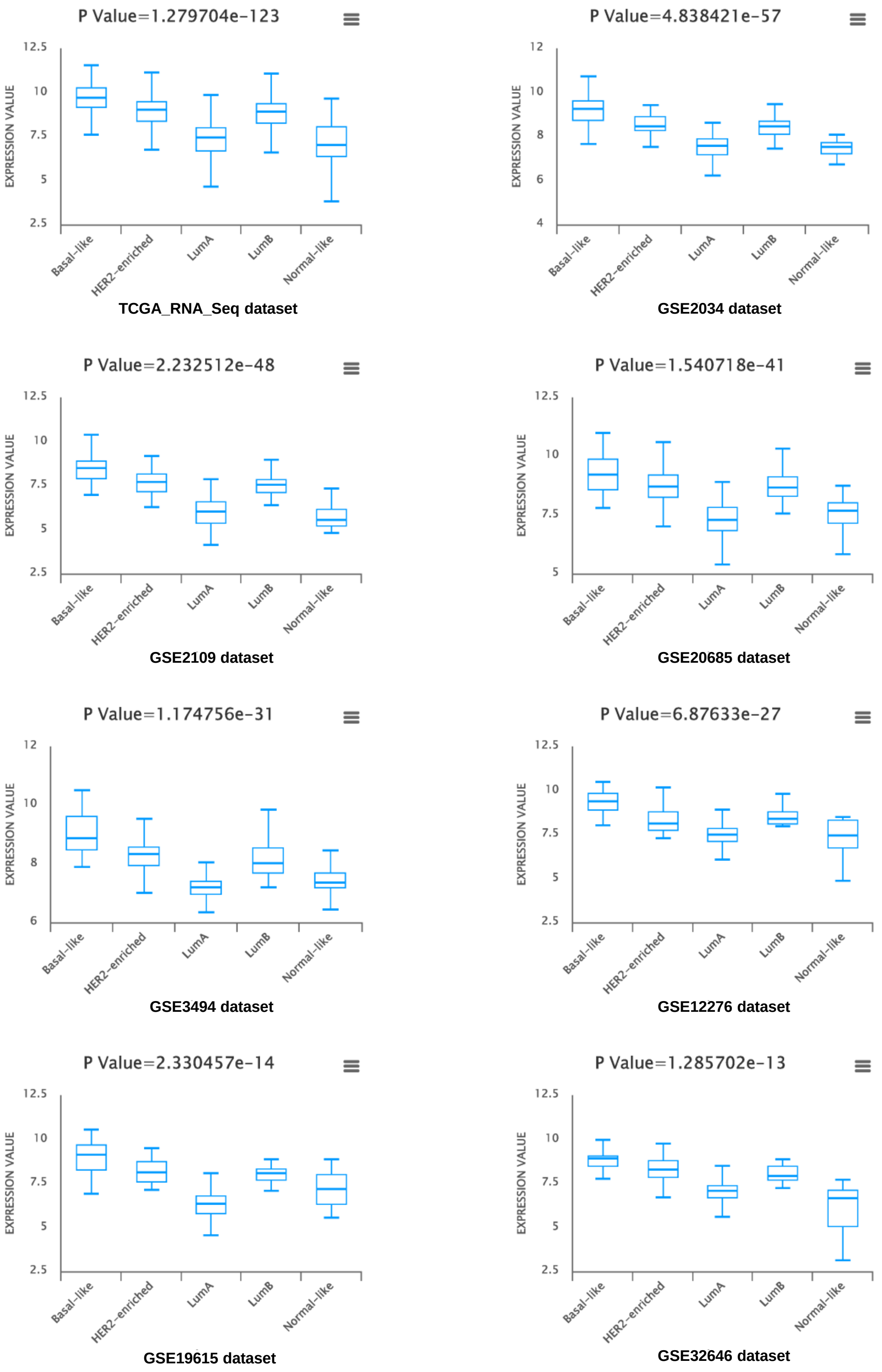

Supplementary Figure S3. Differential expression of *MELK* among PAM50 subtypes. Box plots show the differential expression of *MELK* among PAM50 subtypes in several available datasets, including the TCGA_RNA_Seq, GSE2034, GSE2109, GSE20685, GSE3494, GSE12276, GSE19615, and GSE32646 datasets.

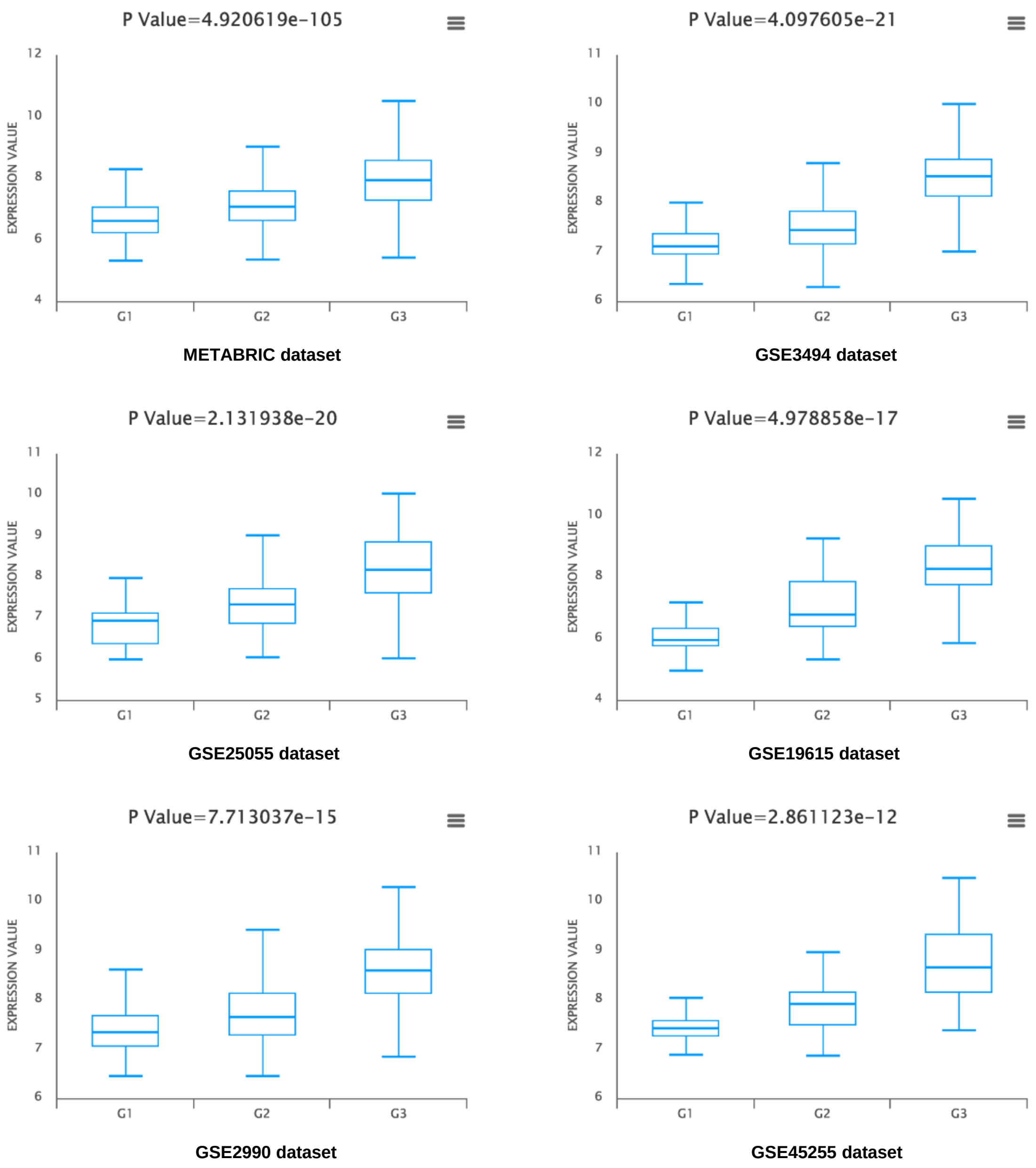

Supplementary Figure S4. Differential expression of *MELK* among 3 histological grades. Box plots show the differential expression of *MELK* among 3 histological grades in several available datasets, including the METABRIC, GSE3494, GSE25055, GSE19615, GSE2990, and GSE45255 datasets.

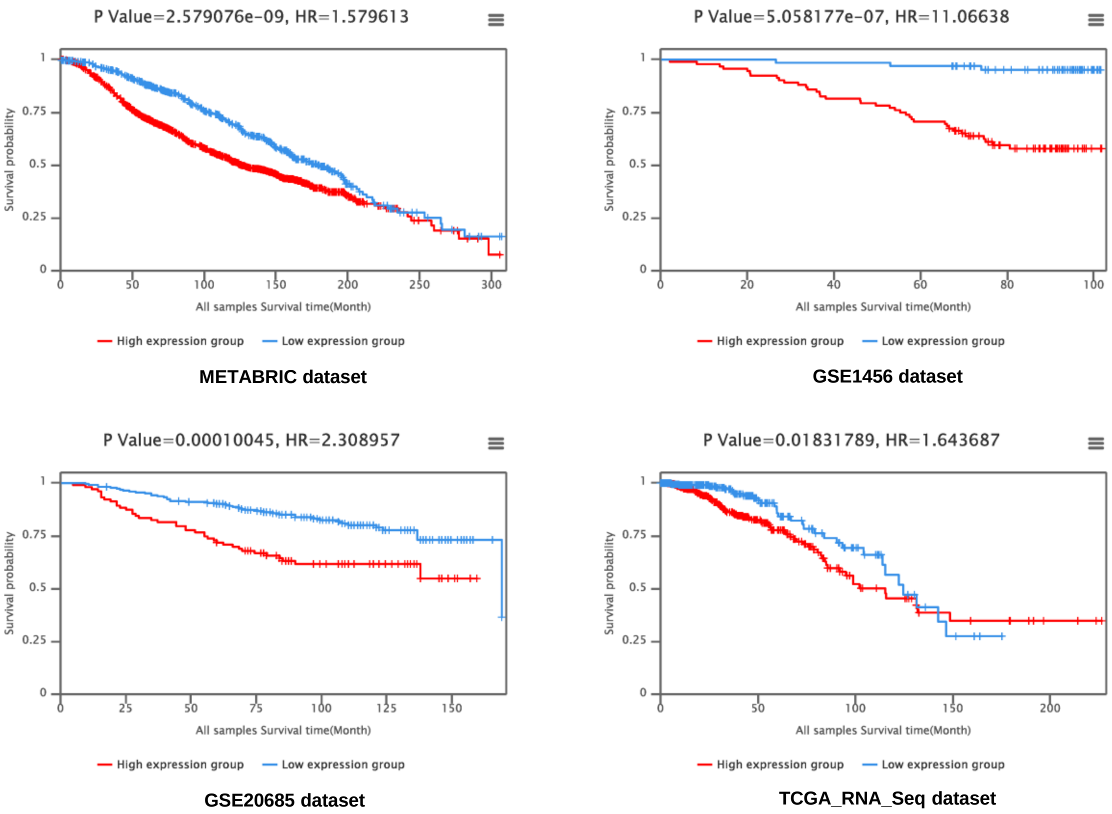

Supplementary Figure S5. Overall survival analysis of patients in several available datasets. Kaplan-Meier plots show the overall survival status between patients with low or high levels of *MELK* expression in the METABRIC, GSE1456, GSE20685, and TCGA_RNA_Seq datasets.

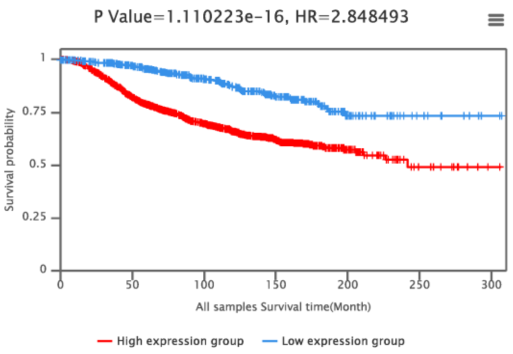

METABRIC dataset

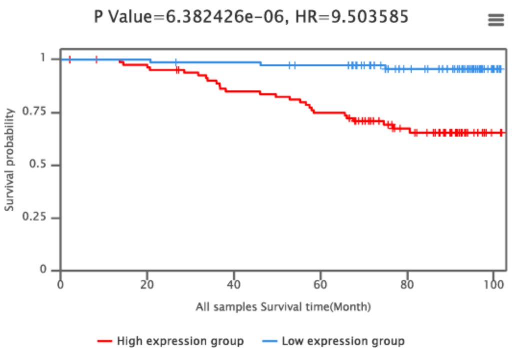

GSE1456 dataset

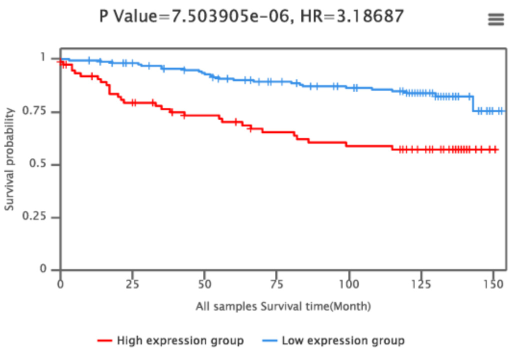

GSE3494 dataset

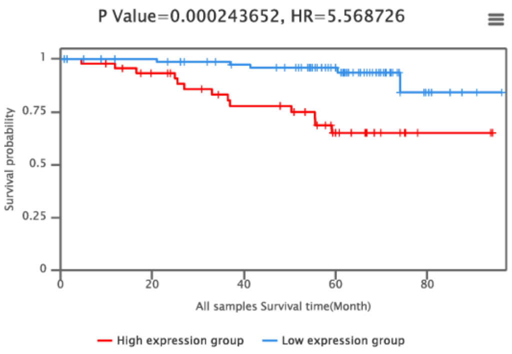

GSE45255 dataset

Supplementary Figure S6. Disease-specific survival analysis of patients across all available datasets. Kaplan-Meier plots show the disease-specific survival status between patients with low or high levels of *MELK* expression in the METABRIC, GSE1456, GSE3494, and GSE45255 datasets.

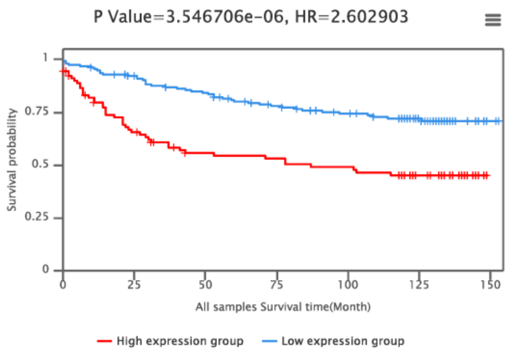

GSE4922 dataset

Supplementary Figure S7. Disease-free survival analysis of patients in the GSE4922 dataset. A Kaplan-Meier plot shows the disease-free survival status between patients with low or high levels of *MELK* expression in the GSE4922 dataset.

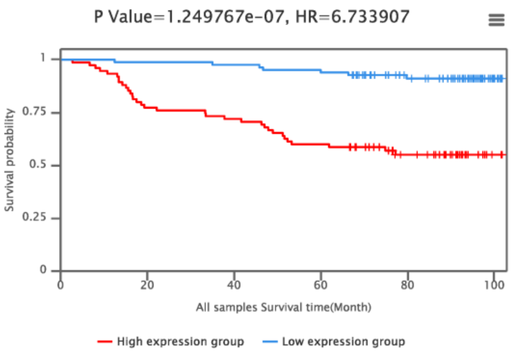

GSE1456 dataset

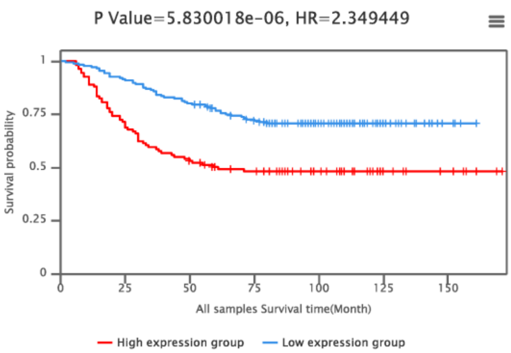

GSE2034 dataset

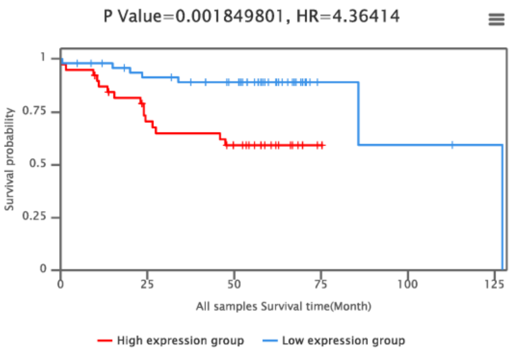

GSE45255 dataset

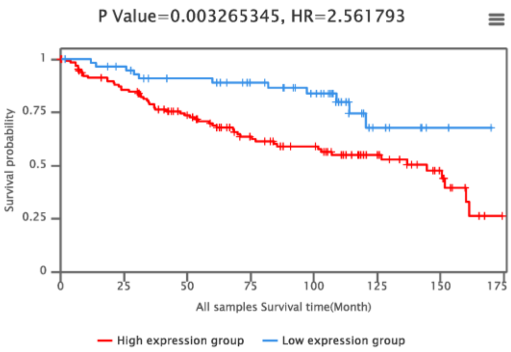

GSE2990 dataset

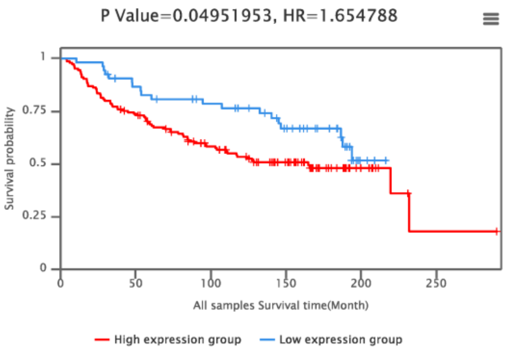

GSE7390 dataset

Supplementary Figure S8. Recurrence-free survival analysis of patients in several available datasets. Kaplan-Meier plots show the recurrence-survival status between patients with low or high levels of *MELK* expression in the GSE1456, GSE2034, GSE45255, GSE2990, and GSE7390 datasets.

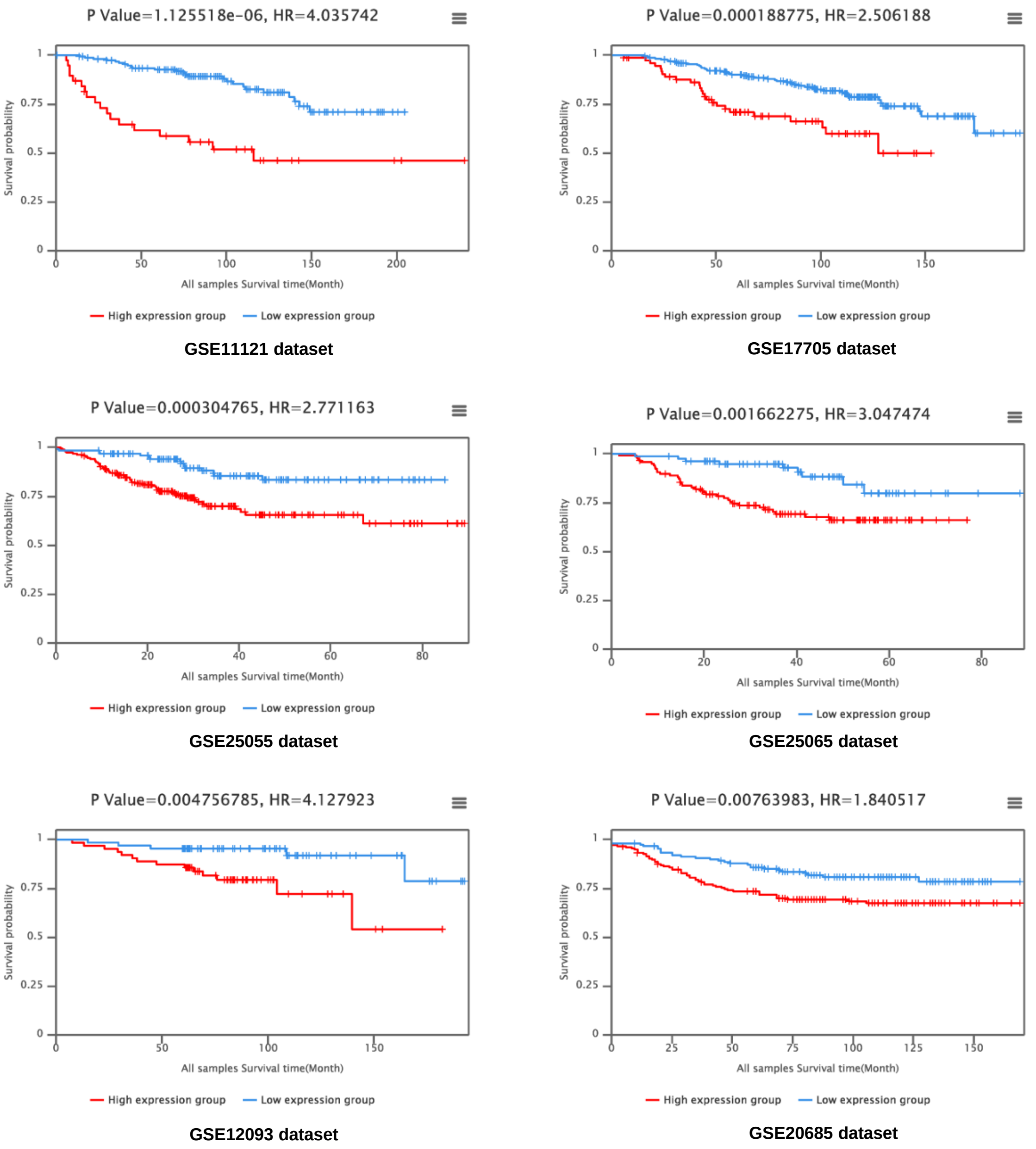

Supplementary Figure S9. Distant metastasis-free survival analysis of patients in several available datasets. Kaplan-Meier plots show the distant metastasis-free survival status between patients with low or high levels of *MELK* expression in the GSE11121, GSE17705, GSE25055, GSE25065, GSE12093, and GSE20685 datasets.
